# Replacing Commercial Substrate with Millicompost: A Sustainable Approach Using Different Green Wastes Combined with Millicompost for Bell Pepper Seedling Production in Urban Agriculture

**DOI:** 10.1021/acsomega.5c06388

**Published:** 2025-09-13

**Authors:** Luiz Fernando de Sousa Antunes, André Felipe de Sousa Vaz, Giulia da Costa Rodrigues dos Santos, Talita dos Santos Ferreira, Renata Rodrigues dos Santos, Renata dos Santos Alves, Jaqueline Carvalho de Almeida, Marco Antonio de Almeida Leal, Maria Elizabeth Fernandes Correia

**Affiliations:** † 74384 Federal Rural University of the Semi-Arid, Mossoró, Rio Grande do Norte 59625900, Brazil; ‡ 67825 Federal Rural University of Rio de Janeiro, Seropédica, Rio de Janeiro 23897-000, Brazil; § Embrapa Agrobiology, Seropédica, Rio de Janeiro 23897-970, Brazil

## Abstract

This study demonstrates that the millicompost is as a sustainable peat-alternative substrate for vegetable seedling production, leveraging its optimal physicochemical properties. We developed seven novel growing media by combining millicompost with regional agro-wastes—coconut coir (*Cocos nucifera*), shavings of the gliricidia (*Gliricidia sepium*), and elephant grass (*Pennisetum purpureum*) for the production of bell pepper seedlings. The substrates formulated were as follows: (S1) millicompost; (S2) Commercial substrate; (S3) 33% millicompost + 33% gliricidia + 33% elephant grass; (S4) 33% millicompost + 33% gliricidia + 33% coconut fiber; (S5) 33% millicompost + 33% elephant grass + 33% coconut fiber; (S6) 25% millicompost + 25% gliricidia + 25% elephant grass + 25% coconut fiber; (S7) 50% millicompost + 50% elephant grass; (S8) 50% millicompost + 50% gliricidia; (S9) 50% millicompost + 50% coconut fiber. Substrates S4 and S8 produced seedlings of a similar quality to millicompost (S1), followed by substrates S3 and S6, which, although they had lower phytotechnical parameters than those mentioned above, have potential for use. The mixture is effective when the substrates are formulated with millicompost, gliricidia and coconut fiber in a ratio (volume/volume) of 33% of each material, respectively, or 50% millicompost + 50% gliricidia. Combining millicompost with vegetable waste allows producers to maximize its use, as well as obtaining quality seedlings and eliminating the need to use commercial substrates.

## Graphical abstract

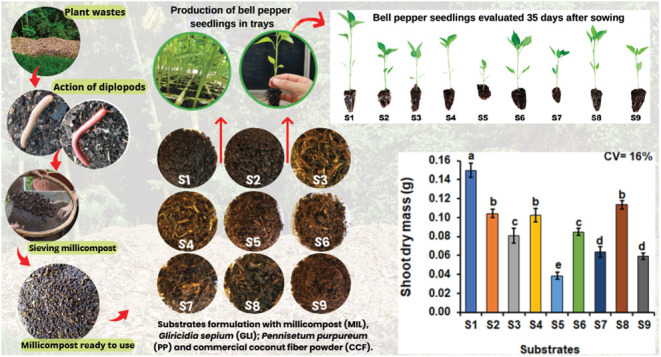

## 1 Introduction

Bell peppers (*Capsicum annuum* L.) belong to the Solanaceae family and are one of the main fruit-type vegetables grown in Brazil. Data from the Agricultural Census shows that the production of peppers in the country amounted to 224,286 tons, ranking eighth among vegetables, with cultivation in all Brazilian regions. The Southeast is the largest producer, accounting for 55.59% of national production, followed by the Northeast (25.52%), South (9.94%), Midwest (6.61%) and North (2.34%).^1^ The texture and visual aspects of the fruit contribute to its excellent marketability, guaranteed by the good practices adopted from sowing to the marketing stage.^2^ Worldwide, peppers are widely used in cooking, whether in salads, culinary ingredients or condiments.^3^

The use of substrates for the production of bell pepper seedlings, as well as other vegetables, is an essential step to ensure greater efficiency and productivity.^4^ Substrates are widely available on the market, but not all of them are known for their efficiency in plant development. Other points to highlight are the need for recurrent fertilization, due to the lack of nutrients suitable for the initial development of seedlings, as well as the use of nonrenewable raw materials such as peat.

Peat is obtained from peatlands, extremely sensitive ecosystems that are home to a rich diversity of organisms, act as important carbon reservoirs and help preserve the quality of groundwater. Due to its negative impact on habitat destruction, peat has been classified by environmentalists as an unsustainable resource. In view of this, there is an urgent demand for studies to expand the options for ecological substrates that can efficiently replace peat.^5^

High-quality substrates are essential for the production of healthy, high-performance seedlings, ensuring successful transplanting to production fields.^6^ Substrates must have physical (such as adequate moisture retention, macroporosity and microporosity), physicochemical (balanced pH and appropriate electrical conductivity), chemical (availability of nutrients, cation exchange capacity and good affinity with roots) and biological (absence of pathogens) properties that favor the development of various species.^7–9^

The nursery phase is a determining factor in obtaining high quality seedlings,^10^ which translates not only into plant health, but also into the robustness of their root system, together reflecting positively on obtaining uniform stands and increases in crop production.^11–13^

Millicompost is an organic substrate from the process of millicomposting, which consists of transforming agricultural waste into stable organic compost—humus from millipedes, whose physical, physicochemical, chemical and biological properties provide the necessary conditions for the development of vegetable seedlings^10,14–17^ and fruit trees,^7,18^ eliminating the need to combine other materials to make up a substrate, as is done in the production of commercial substrates, since it is ready to use.^19^

Using millicompost as a substrate in seedling production systems not only reduces dependence on chemical inputs and commercial substrates, but also contributes to reducing the carbon footprint, since the controlled decomposition of agricultural and urban pruning waste prevents the emission of greenhouse gases associated with the improper disposal of organic matter. In addition, millicomposting strengthens food security by providing high-quality substrates for the production of vegetable and fruit seedlings, ensuring more resilient and accessible production systems, especially for family farmers.

These practices are in line with the United Nations Sustainable Development Goals (SDGs),^20^ especially SDG 2 (Zero Hunger and Sustainable Agriculture), SDG 11 (Sustainable Cities and Communities), 12 (Responsible Consumption and Production) and SDG 13 (Action against Global Climate Change). By transforming waste into resources, millicomposting promotes environmental, economic and social sustainability, reinforcing its role as a green and inclusive technology.

Although millicompost does not require mixing with other raw materials, the yield of millicomposting is approximately 30%, since the vegetable waste supplied is senescent (street and park sweeping leaves, dried grass clippings, banana leaves, crop residues, etc.) and when combined together makes up a large volume, which is reduced by up to 70% during the process, which can last at least 120 days.^21^

Considering that the amount of millicompost produced may not be enough to meet the demand of seedling producers, this study proposes investigating components which, when combined in different proportions with millicompost, can provide results as efficient as those obtained using millicompost alone. To this end, this study tested different formulations of organic substrates based on millicompost, combined with varying proportions of powdered coconut fiber, gliricidia shavings and elephant grass. Our hypothesis is that the formulated substrates have comparable efficiency to pure millicompost in the development of seedlings. To test this hypothesis, bell pepper seedlings were grown in 128-cell expanded polystyrene trays under controlled greenhouse conditions.

## 2 Material and Methods

### 2.1 Production of Millicompost

The millicompost, used as the basis for formulating the substrates, was produced in an open environment near a small area of secondary vegetation in the Atlantic Forest Biome in the state of Rio de Janeiro, located in the southeast of Brazil. The waste added to the millicomposting process came from tree pruning and garden trimmings, as described by Antunes et al.^7^ and the millipedes (diplopods) belonging to the species *Trigoniulus corallinus*,^22^ native to Southeast Asia, easily found in anthropized areas, rich in organic and moist materials^23^ and *Rhinocricus padbergi*,^24^ native to the Brazilian Atlantic Forest, known as the giant millipede.

### 2.2 Formulation of Substrates

The organic substrates were formulated from renewable organic sources: commercial powdered coconut fiber (CCF), leaves and stems of *Gliricidia sepium* (GLI) and *Pennisetum purpureum* (PP). The latter two were chopped and passed through the chipper to obtain particles of ± 2 cm and then spread individually in a shed, forming a layer of 10 cm, kept in the shade for 30 days. They were turned over every 10 days to speed up the drying process. The pH values, electrical conductivity (EC), C/N ratio and nutrient content of the residues are contained in Table 1. Although commercial coconut fiber was used for this study, all of these residues can be obtained locally on the farm.

**Table 1:** pH, Electrical Conductivity (EC), C/N Ratio and Levels of Macronutrients Present in the Residues Used to Formulate Different Substrates with the Addition of Millicompost^a^

organic wastes	pH	EC (dS m^–1^)	C/N ratio	N (g kg^–1^)	P (g kg^–1^)	K (g kg^–1^)	Ca (g kg^–1^)	Mg (g kg^–1^)
*G. sepium*	9.2	1.0	30	13.4	1.8	18.6	12.5	2.8
*P. purpureum*	11.1	1.5	36	9.9	4.1	32.1	3.9	1.6
coconut fiber powder	5.9	0.9	63	6.4	4.6	12.3	12.0	2.9

a pH and EC values obtained according to the methodology described by Brasil^25^ and total nutrients by Teixeira et al.^26^

The substrate formulations were made on a volume/volume (v/v) basis, which were quantified and added to a concrete mixer for better homogenization and subsequent use. The nine substrates used were: S1—Millicompost; S2—Commercial substrate (based on composted pine bark); S3–33% Millicompost + 33% GLI + 33% PP; S4–33% Millicompost + 33% GLI + 33% CCF; S5–33% Millicompost + 33% PP + 33% CCF; S6–25% Millicompost + 25% GLI + 25% PP + 25% CCF; S7–50% Millicompost + 50% PP; S8–50% Millicompost + 50% GLI and S9–50% Millicompost + 50% CCF.

### 2.3 Analysis of the Physical, Physicochemical, and Chemical Properties of the Substrates

The physical characteristics assessed in the substrates were as follows: macroporosity, microporosity, total porosity, water retention capacity and volumetric density.^26^ Aluminum cylinders with a volumetric capacity of 100 cm^3^ were used, with the bottom opening covered with nonwoven fabric (TNT). The cylinders were placed on a drainage setup, with their bases in contact with blotting paper. The blotting paper had pore diameters ≤ 0.0025 cm (2.5 μm). Additionally, care was taken to prevent trapped air between the blotting paper and the underlying system. The setup remained under a suction pressure equivalent to a 60 cm water column (0.06 bar tension) for 24 h. After drainage, the cylinders containing the substrates were transferred to an oven at 105 °C. Following 48 h of drying, they were weighed. The following formulas were used to determine the physical properties

1 $$\mathrm{macroporosity}\;\left(\%\right)=\left[\frac{\left(A-B\right)}{C}\right]\times 100$$

2 $$\mathrm{microporosity}\;\left(\%\right)=\left[\frac{\left(B-D-E\right)}{C}\right]\times 100$$

3 totalporosity(%)=[macroporosity+microporosity]×100

4 $$w\mathrm{ater retention capacity}\;\left(mL\;50\;{cm}^{-3}\right)=B-D-E$$

5 $$\mathrm{volumetric}\;\mathrm{density}\;\left(g\;{\mathrm{cm}}^{-3}\right)=\frac{D-E}{C}$$

Where: *A* = weight of saturated substrate; *B* = weight of drained substrate; *C* = volume of the container; *D* = weight of oven-dried substrate; *E* = weight of the container.

In order to characterize the substrates in terms of their physical and chemical characteristics, pH analyses were carried out in a distilled water solution (5:1 v/v) and electrical conductivity was determined in the same aqueous extract obtained for the pH measurement, according to the method described by Brasil.^25^ As for the chemical properties, samples of each organic substrate were sent to to the Agricultural Chemistry Laboratory at Embrapa Agrobiologia to determine the N, P, K, Ca, and Mg contents, according to the methodology described by Teixeira et al.^26^

With the exception of chemical parameters, all analyses related to physical and physicochemical properties were performed in triplicate.

### 2.4 Production Bell Pepper Seedlings Grown in Different Substrates and Morphological Evaluations

The experiment was conducted in a greenhouse from June 27 to August 1, 2019, located on the premises of the Brazilian Agricultural Research Corporation (Embrapa Agrobiologia), in the municipality of Seropédica, state of Rio de Janeiro, in the Southeast region of Brazil. The altitude of the site is 33.0 m above sea level and the climate is classified as tropical humid (Aw), with rainfall concentrated in the period from November to March, average annual rainfall of 1213 mm and average annual temperature of 24.5 °C.^27^

Expanded polystyrene trays with 128 cells were used to sow ISLA Cascadura Ikeda green beel peppers (*Capsicum annuum* L.), with two seeds per cell. Ten days after sowing, the tray was thinned out, leaving only one plant per cell. At 35 days after sowing, ten bell pepper seedlings per experimental unit (tray) were taken at random and the following phytotechnical parameters were assessed: shoot dry mass, root dry mass, plant height, which includes the point of root insertion up to the leaf apex, number of leaves, seedling vigor and clod stability. To determine the dry masses, the aerial part and the roots of the plants were packed separately in paper bags and kept in a forced-air oven at 65 °C for 72 h, after which their weights were measured on a precision scale (0.01 g).

Seedling vigor is a methodology adapted from Antunes et al.^11^ classifying it as Note 1: excellent vigor, number of leaves ≥ 4, height ≥ 10 cm, presence of cotyledonary leaves and visual absence of nutritional deficiency; Note 2: good vigor, number of leaves ≥ 4, height ≥ 10 cm and nonprominent yellowing of cotyledonary or basal leaves; Note 3: regular vigor, number of leaves ≥ 4, height ≥ 5 cm; nutritional deficiency expressed by prominent yellowing extending beyond the cotyledonary or basal leaves and/or another intrinsic symptom; Note 4: poor vigor, very prominent nutritional deficiency expressed by problems with seedling height (<5 cm), reduced number of leaves (<4 leaves) and intense yellowing and/or another intrinsic symptom.

Clod stability is a methodology adapted from Antunes et al.^11^ classifying it as Note 1: Low stability, 50% or more of the clod is retained in the container when the seedling is removed and the clod does not remain cohesive; Note 2: Between 30 and 50% of the clod is retained in the container when the seedling is removed, but the clod does not remain cohesive; Note 3: Fair, between 15 and 30% of the clod is retained in the container when the seedling is removed, but does not remain cohesive; Note 4: Good stability, the clod is detached completely from the container with up to 90% cohesion and a maximum loss of up to 10% of the substrate; Note 5: Excellent stability, the clod is detached completely from the container and more than 90% of it remains cohesive, with losses of less than 10% of the substrate.

### 2.5 Statistical Analysis

The experimental design adopted was randomized blocks with five replications (trays) and nine treatments (substrates). For the statistical analysis of the data generated, the homogeneity of the error variances was checked using the Bartlett test and normality using the Shapiro–Wilk test. Subsequently, the data was subjected to analysis of variance using the Scott–Knott test at a 5% probability level, using the Rbio statistical program.^28^

Principal Component Analysis (PCA) and correlation analyses were performed in the RStudio environment (v2023.03.0) using the factoextra package (v1.0.7) for PCA visualization, ggcorrplot (v0.1.4.1) for interactive correlation matrices, and tidyverse (v1.3.1) for data manipulation, all implemented in R (v4.1.2).^29^ For the clustering analysis, the performance data of the nine substrates (S1–S9) were standardized using *Z*-scores and analyzed through agglomerative hierarchical clustering, employing squared Euclidean distance as a dissimilarity measure and Ward’s linkage method for intragroup variance minimization. The robustness of the dendrogram was assessed by the cophenetic correlation (*r* = 0.89), with three main clusters defined through aggregation coefficient analysis. Differences between groups were validated by univariate ANOVA (*p* < 0.05) with Tukey’s posthoc test, while visualization was performed using the ggplot2 package in R 4.3.1, representing the clusters in a chromatic gradient (red-orange-green) according to performance.

## 3 Results

### 3.1 Physical Properties of Substrates Organic

The percentage of macropores differed between the substrates, with the highest percentage recorded for the Millicompost substrate (S1), followed by S2, which had the second highest percentage. Substrates S4, S5, S6 and S9 showed the lowest results (Table 2).

**Table 2:** Physical Analysis of the Substrates Used in the Production of Bell Pepper (*C. annuum* L.) Seedlings: Percentages of Macroporosity, Microporosity, Total Porosity, Water Retention Capacity at 10 cm (WRC 10 cm) and Volumetric Density, with Their Respective Standard Errors^a^

substrates	macroporosity (%)	microporosity (%)	total porosity (%)	WRC_10cm_ (mL 50 cm^–3^)	volumetric density (g m^–3^)
S1	43.45 ± 4.5	39.64 ± 2.8	83.09 ± 3.0	37.14 ± 0.9	0.40 ± 0.0
S2	27.48 ± 28.1	58.33 ± 34.7	85.81 ± 6.8	35.55 ± 12.0	0.21 ± 0.0
S3	19.34 ± 4.2	60.59 ± 3.3	79.93 ± 6.1	36.32 ± 1.4	0.17 ± 0.0
S4	7.50 ± 1.1	75.56 ± 0.8	83.06 ± 1.1	38.99 ± 0.8	0.18 ± 0.0
S5	7.50 ± 0.0	76.27 ± 7.0	83.77 ± 7.0	39.35 ± 3.5	0.20 ± 0.0
S6	8.84 ± 0.7	80.75 ± 3.7	89.59 ± 3.7	41.18 ± 2.0	0.19 ± 0.0
S7	15.36 ± 5.4	75.56 ± 4.4	90.92 ± 6.4	42.67 ± 2.6	0.27 ± 0.0
S8	18.07 ± 3.7	69.48 ± 3.3	87.55 ± 4.0	39.41 ± 2.1	0.25 ± 0.0
S9	10.11 ± 2.0	61.16 ± 1.6	71.27 ± 1.6	30.84 ± 0.6	0.18 ± 0.0

a S1—Millicompost; S2—Commercial substrate; S3–33% MIL + 33% GLI + 33% PP; S4—33% MIL + 33% GLI + 33% CCF; S5—33% MIL + 33% PP + 33% CCF; S6—25% MIL + 25% GLI + 25% PP + 25% CCF; S7—50% MIL + 50% PP; S8— 50% MIL + 50% GLI; S9—50% MIL + 50% CCF. MIL: Millicompost; GLI: *G. sepium*; PP: *P. purpureum*; CCF: commercial coconut fiber powder.

As for microporosity, the results were basically the inverse of macroporosity, with substrate S1 showing the lowest percentage of micropores. As for total porosity, the highest percentages were found in substrates S6, S7, and S8 and the lowest in S9 (Table 2). As for water retention capacity (WRC), the highest values were found in S4, S5, S6, S7, and S8 and the lowest in substrate S9. Substrate S1 had the highest volumetric density and S3, S4, S6, and S9 had the lowest values (Table 2).

### 3.2 Physico-Chemical Properties of Organic Substrates

All the substrates, with the exception of S2 (commercial), had pH values above 5, and substrates S6, S7, and S8 had values above 8 (Table 3). As for electrical conductivity (EC), there was also variation between the substrates, where the highest values were found in substrates S3, S7, and S8 (Table 3).

**Table 3:** Physico-Chemical Analysis of the Substrates Used to Produce Bell Pepper (*C. annuum* L.) Seedlings, with Their Respective Standard Errors^a^

substrates	pH	EC (dS m^–1^)
S1	5.87 ± 0.6	0.94 ± 0.0
S2	4.64 ± 0.2	0.39 ± 0.0
S3	7.90 ± 1.4	1.10 ± 0.1
S4	7.42 ± 0.2	0.75 ± 0.2
S5	7.93 ± 0.2	0.60 ± 0.2
S6	8.19 ± 0.1	0.81 ± 0.0
S7	8.78 ± 0.2	0.99 ± 0.0
S8	9.06 ± 0.1	1.06 ± 0.0
S9	6.50 ± 0.6	0.66 ± 0.0

a S1—Millicompost; S2—Commercial substrate; S3–33% MIL + 33% GLI + 33% PP; S4—33% MIL + 33% GLI + 33% CCF; S5—33% MIL + 33% PP + 33% CCF; S6—25% MIL + 25% GLI + 25% PP + 25% CCF; S7—50% MIL + 50% PP; S8—50% MIL + 50% GLI; S9—50% MIL + 50% CCF. MIL: Millicompost; GLI: *G. sepium*; PP: *P. purpureum*; CCF: commercial coconut fiber powder.

### 3.3 Chemical Properties of Organic Substrates

The nutritional characterization of substrates assessed total concentrations, plant-available forms, and their proportional relationships for five essential macronutrients (N–P–K–Ca–Mg), as detailed in Table 4. The N content, which is generally the nutrient most demanded by seedlings, ranged from 10.41 g kg^–1^ (S2) to 16.56 g kg^–1^ (S1). As for the phosphorus (P) and potassium (K) nutrient levels, the S3 substrate had the highest values, while the lowest P contents were observed for millicompost (S1) and K for commercial substrate (S2). In terms of calcium (Ca) content, only the commercial substrate (S2) was lower than the other substrates. The magnesium (Mg) content ranged from 1.24 (S2) to 4.75 g kg^–1^ (S8).

**Table 4:** Chemical Analysis of Total and Available Nutrients in the Substrates Used to Produce Bell Pepper (*C. annuum* L.) Seedlings^a^

total contents					
substrates	N (g kg^–1^)	P (g kg^–1^)	K (g kg^–1^)	Ca (g kg^–1^)	Mg (g kg^–1^)
S1	16.56	1.57	3.17	20.15	3.23
S2	10.41	2.15	2.46	5.23	1.24
S3	16.33	2.74	14.85	24.11	3.27
S4	14.63	1.91	10.08	18.77	2.54
S5	13.17	2.24	10.90	19.11	2.43
S6	13.91	2.45	14.24	17.65	2.67
S7	14.87	2.43	10.75	22.81	3.52
S8	17.32	2.22	7.93	26.31	4.75
S9	13.15	1.71	7.09	19.42	2.92

available contents					
substrates	N (g kg^–1^)	P (g kg^–1^)	K (g kg^–1^)	Ca (g kg^–1^)	Mg (g kg^–1^)
S1	2.25	0.81	2.80	12.69	2.72
S2	3.88	1.57	2.02	4.78	1.06
S3	3.33	1.97	14.27	9.27	2.03
S4	3.30	1.50	10.08	10.54	2.42
S5	2.62	1.67	10.90	10.09	1.90
S6	2.35	1.76	12.88	9.88	2.04
S7	2.37	2.06	10.75	10.39	1.92
S8	2.72	1.50	7.93	11.42	2.32
S9	2.55	1.37	7.09	11.81	2.20

proportion of available nutrient					
substrates	N (%)	P (%)	K (%)	Ca (%)	Mg (%)
S1	13.56	51.88	88.45	62.98	84.26
S2	37.29	73.19	82.22	91.33	85.25
S3	20.39	71.91	96.09	38.43	62.05
S4	22.56	78.39	100.00	56.14	95.19
S5	19.92	74.67	100.00	52.78	78.00
S6	16.92	71.70	90.43	55.98	76.45
S7	15.93	84.85	100.00	45.57	54.54
S8	15.71	67.78	100.00	43.40	48.86
S9	19.41	79.97	100.00	60.80	75.31

a S1—Millicompost; S2—Commercial substrate; S3–33% MIL + 33% GLI + 33% PP; S4—33% MIL + 33% GLI + 33% CCF; S5—33% MIL + 33% PP + 33% CCF; S6—25% MIL + 25% GLI + 25% PP + 25% CCF; S7—50% MIL + 50% PP; S8—50% MIL + 50% GLI; S9—50% MIL + 50% CCF. MIL: Millicompost; GLI: *G. sepium*; PP: *P. purpureum*; CCF: commercial coconut fiber powder.

Regarding the available forms of nutrients, it was observed that nitrogen (N) showed greater availability in the commercial substrate S2 (37.29% of the total), while the lowest percentage was recorded in S1 (13.56%). Phosphorus (P) stood out for its high proportion of availability in all substrates (51.88–84.85%), with emphasis on S7 (84.85%). Potassium (K), calcium (Ca), and magnesium (Mg) exhibited distinct patterns: K reached 100% availability in five substrates (S4, S5, S7, S8, S9), Ca showed greater availability in S2 (91.33%) and Mg in S4 (95.19%).

As for the carbon/nitrogen ratio (C/N), it can be seen that substrate S2 had a higher C/N ratio than the others, while S8 had the lowest value (Table 5). The C content varied from 27% (S7) to 38% (S2) (Table 5).

**Table 5:** Quantification of Organic Carbon and C/N Ratio of the Substrates for Bell Pepper (*C. annuum* L.) Seedling Cultivation^a^

substrates	C/N ratio	organic carbon (%)
S1	18.91	35.00
S2	36.86	38.37
S3	17.67	28.84
S4	22.07	32.29
S5	23.97	31.56
S6	24.17	33.61
S7	18.74	27.85
S8	17.07	29.57
S9	23.78	31.26

a S1—Millicompost; S2—Commercial substrate; S3–33% MIL + 33% GLI + 33% PP; S4—33% MIL + 33% GLI + 33% CCF; S5—33% MIL + 33% PP + 33% CCF; S6—25% MIL + 25% GLI + 25% PP + 25% CCF; S7—50% MIL + 50% PP; S8— 50% MIL + 50% GLI; S9—50% MIL + 50% CCF. MIL: Millicompost; GLI: *G. sepium*; PP: *P. purpureum*; CCF: commercial coconut fiber powder.

### 3.4 Morphological Parameters of Bell Pepper Seedlings

The organic substrates formulated from millicompost produced bell pepper seedlings with significantly different morphological characteristics (*p* ≤ 0.05) for all the phytotechnical parameters evaluated (Figure 1). In terms of shoot dry mass, the highest average was observed in substrate S1 (millicompost) and the lowest was recorded for substrate S5 (33% MIL + 33% PP + 33% CCF) (Figure 1A). The substrates S2 (commercial), S4 (33% MIL + 33% GLI + 33% CCF) and S8 (50% MIL + 50% GLI) were the ones that showed similar averages, being higher when compared to the substrates S3 (33% MIL + 33% GLI + 33% PP), S6 (25% MIL + 25% GLI + 25% PP + 25% CCF), S7 (50% MIL + 50% PP) and S9 (50% MIL + 50% CCF) (Figure 1A).

**Figure 1:**
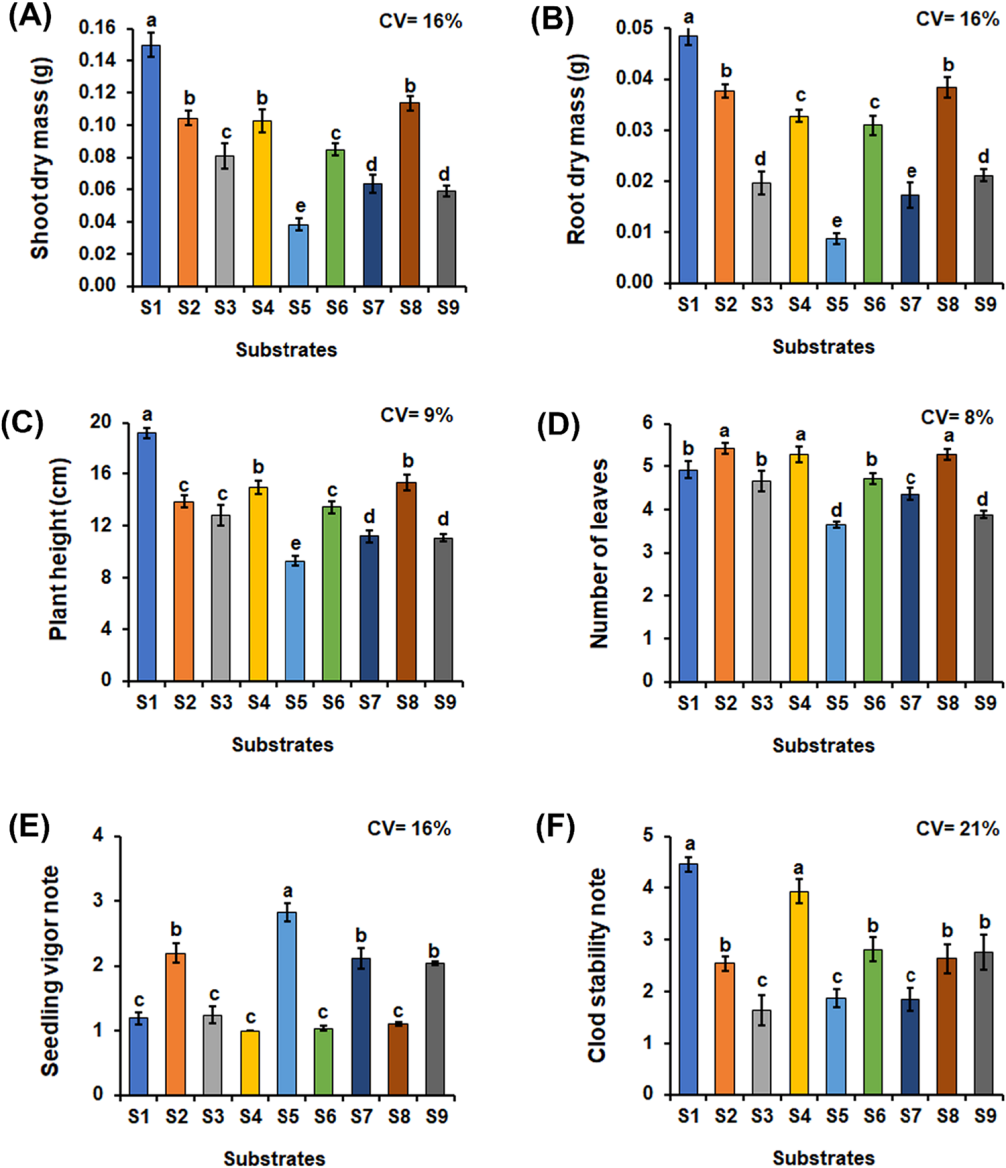
Average values for shoot dry mass (A); root dry mass (B), plant height (C), number of leaves (D), seedling vigor (E) and clod stability (F) of bell pepper (*C. annuum* L.) seedlings produced with different substrate formulations and evaluated 35 days after sowing. Means followed by the same letter in the bars do not differ from each other by the Scott–Knott test (*p* ≤ 0.05). Substrates: S1—Millicompost; S2—Commercial substrate; S3–33% MIL + 33% GLI + 33% PP; S4—33% MIL + 33% GLI + 33% CCF; S5—33% MIL + 33% PP + 33% CCF; S6—25% MIL + 25% GLI + 25% PP + 25% CCF; S7—50% MIL + 50% PP; S8—50% MIL + 50% GLI; S9—50% MIL + 50% CCF. MIL: Millicompost; GLI: Gliricidia sepium; PP: Pennisetum purpureum; CCF: commercial coconut fiber powder.

The highest average root dry mass, as well as the shoot dry mass, was provided by substrate S1 and the lowest by S5 and S7 (Figure 1B). As for the height of the seedlings, the substrate that provided the highest average was S1, followed by substrates S4 and S8 and the lowest in plants grown in substrate S5 (Figure 1C). The number of leaves varied between the treatments, of which S2, S4, and S8 had the most (Figure 1D).

Substrates S1, S3, S4, S6, and S8 produced seedlings with excellent vigor (Figures 1E and 2). Substrates S2, S7, and S9 resulted in seedlings considered to be of good vigor, while S5 was the only one that produced seedlings of fair vigor. None of the substrates produced seedlings of poor vigor. For clod stability, there was also a statistical difference between the substrates, with S1 and S4 being the substrates with the most stable clods, followed by S2, S6, S8, and S9. On the other hand, substrates S3, S5, and S7 showed the lowest stability (Figures 1F and 2).

**Figure 2:**
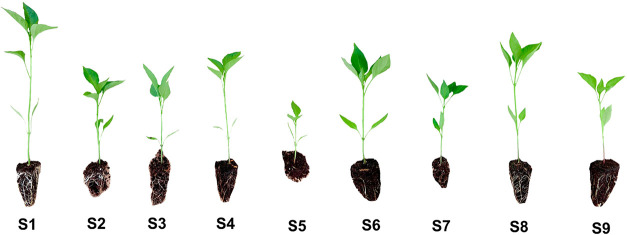
Clear differences between bell pepper (*C. annuum* L.) seedlings produced with different substrate formulations at 40 days after sowing. S1—Millicompost; S2—Commercial substrate; S3–33% MIL + 33% GLI + 33% PP; S4—33% MIL + 33% GLI + 33% CCF; S5—33% MIL + 33% PP + 33% CCF; S6—25% MIL + 25% GLI + 25% PP + 25% CCF; S7—50% MIL + 50% PP; S8—50% MIL + 50% GLI; S9—50% MIL + 50% CCF. MIL: Millicompost; GLI: *G. sepium*; PP: *P. purpureum*; CCF: commercial coconut fiber powder. Photograph courtesy of author Luiz Fernando de Sousa Antunes. Copyright 2025.

Principal Component Analysis (PCA) revealed clear differentiation in substrate performance. Substrate S4 exhibited the most distinct profile, positioned at the far left of PC1, indicating superior performance in biomass accumulation and plant height. In contrast, substrates S5, S7, and S9 clustered on the opposite side of PC1, demonstrating the lowest vegetative vigor (SV) in bell pepper seedlings (Figure 3).

**Figure 3:**
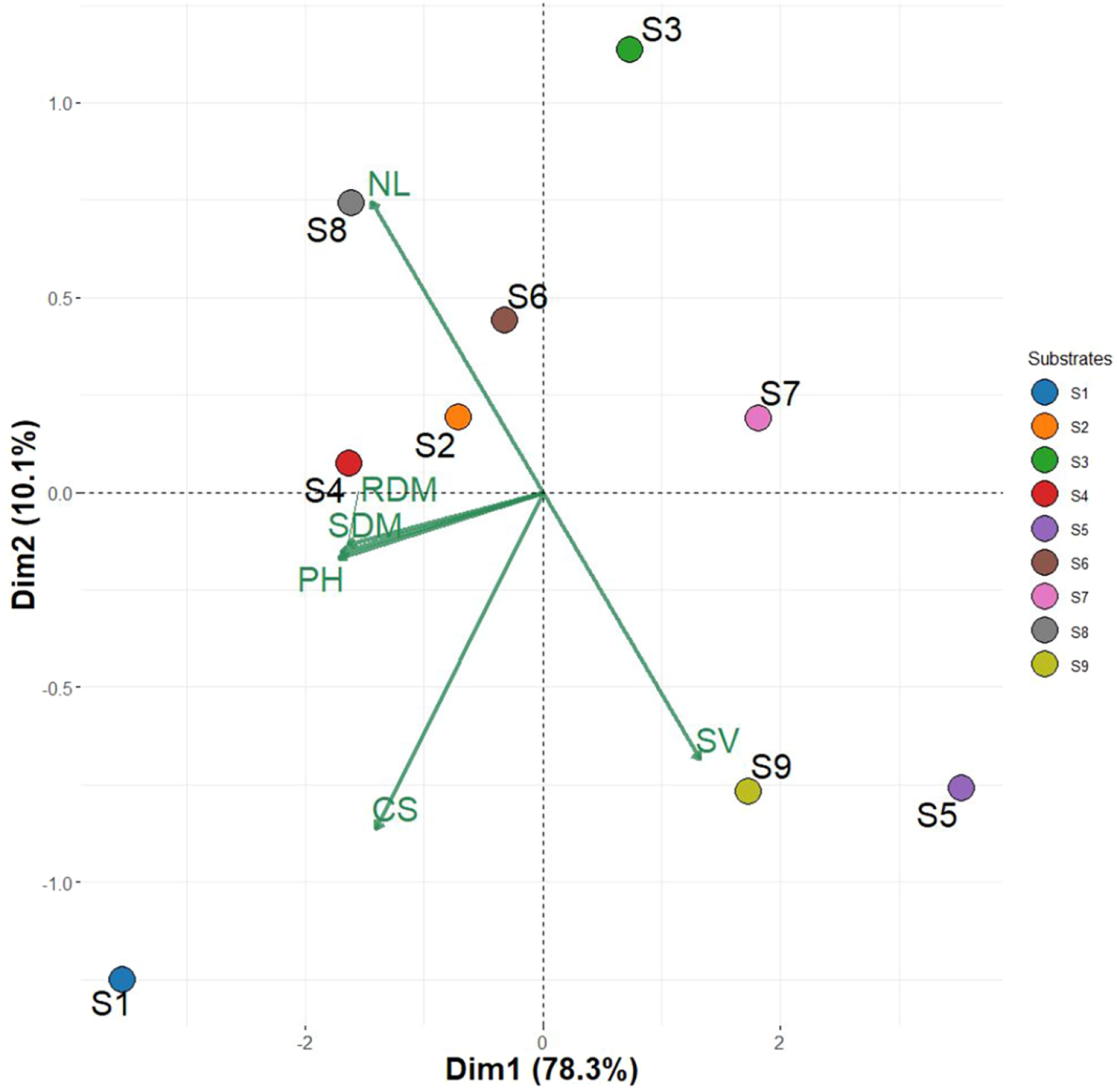
Principal component analysis (PCA) of morphological traits in bell pepper (*C. annuum* L.) seedlings grown in millicompost-based organic substrates. Evaluated variables: shoot dry mass (SDM), root dry mass (RDM), plant height (PH), number of leaves (NL), seedling vigor (SV), and clod stability (CS). Substrates (S1–S9) were standardized by *Z*-score prior to hierarchical clustering using squared Euclidean distance and Ward’s linkage method (cophenetic correlation = 0.89). Three distinct clusters were identified through aggregation coefficient analysis and validated by one-way ANOVA (*p* < 0.05) with Tukey’s posthoc test. S1—Millicompost; S2—Commercial substrate; S3–33% MIL + 33% GLI + 33% PP; S4—33% MIL + 33% GLI + 33% CCF; S5—33% MIL + 33% PP + 33% CCF; S6—25% MIL + 25% GLI + 25% PP + 25% CCF; S7—50% MIL + 50% PP; S8—50% MIL + 50% GLI; S9—50% MIL + 50% CCF. MIL: Millicompost; GLI: *G. sepium*; PP: *P. purpureum*; CCF: commercial coconut fiber powder.

Growth parameters and substrate physical, physicochemical, and chemical properties showed significant relationships with shoot dry mass (SDM) and root dry mass (RDM). These parameters were positively correlated with macroporosity (*r* > 0.7, *p* < 0.05) and negatively associated with microporosity (*r* < −0.6), suggesting that substrates with better aeration promote biomass accumulation. Plant height (PH) showed a nonsignificant positive trend with total porosity (*r* = 0.14, *p* > 0.05) but a significant negative correlation with potassium content (*r* = −0.51, *p* < 0.05), indicating that both physical structure and potassium availability drive seedling growth. In contrast, leaf number (NL) and clod stability (CS) showed weak correlations with chemical properties (pH, EC), implying these traits are less dependent on substrate chemistry. Notably, substrate volumetric density exhibited strong positive correlations with most growth parameters (*r* > 0.5), demonstrating that high density severely limits seedling development (Figure 4).

**Figure 4:**
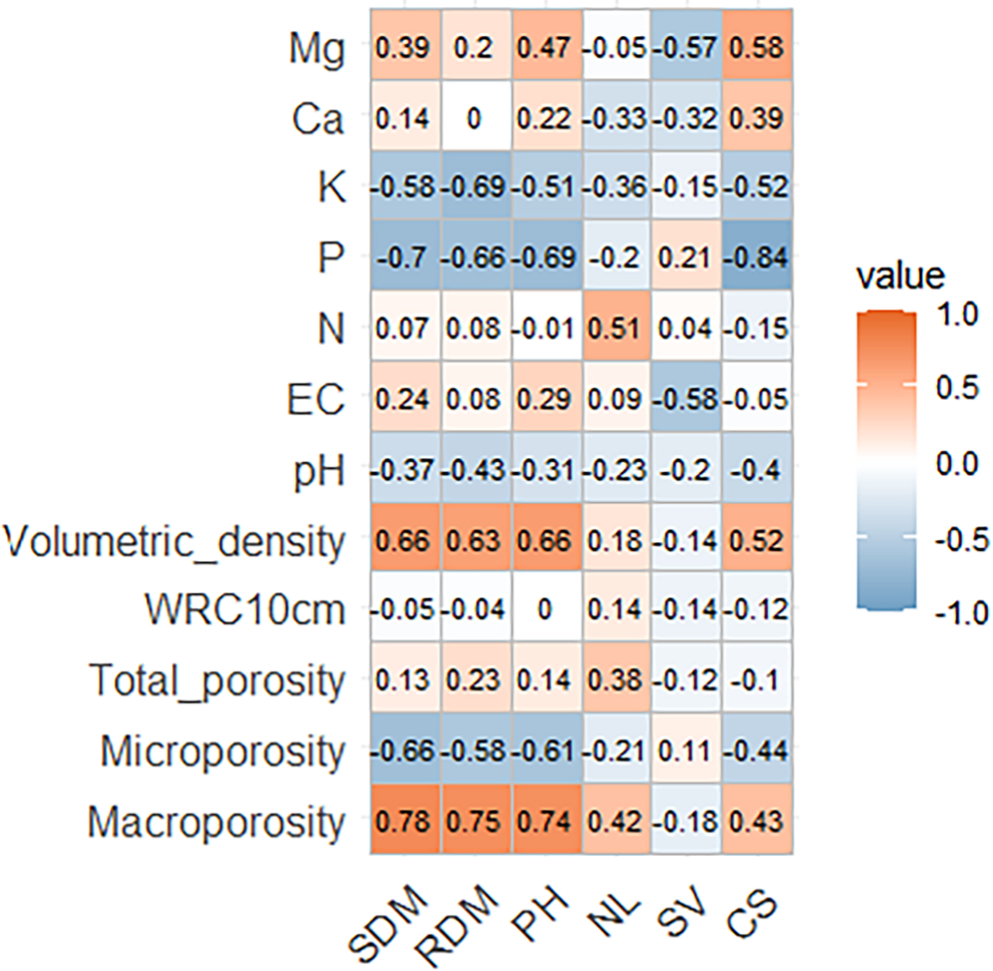
Correlation matrix of growth parameters of pepper seedlings (*C. annuum* L.) [Shoot dry mass (SDM), root dry mass (RDM), plant height (PH), number of leaves (NL), seedling vigor (SV), and clod stability (CS)] and physical, physicochemical and chemical characteristics of the substrates.

Hierarchical cluster analysis of substrate performance revealed three distinct groups (Figure 5). The high-performance cluster (S4, S8) showed superior shoot biomass (SDM = 0.10–0.11 g) and plant height (PH = 14–15 cm), while S1 demonstrated maximum SDM (0.15 g) and clod stability (CS = 4.46). Substrates S2, S6, and S3 exhibited intermediate growth characteristics, whereas substrates S5, S7, and S9 resulted in compromised development of bell pepper seedlings.

**Figure 5:**
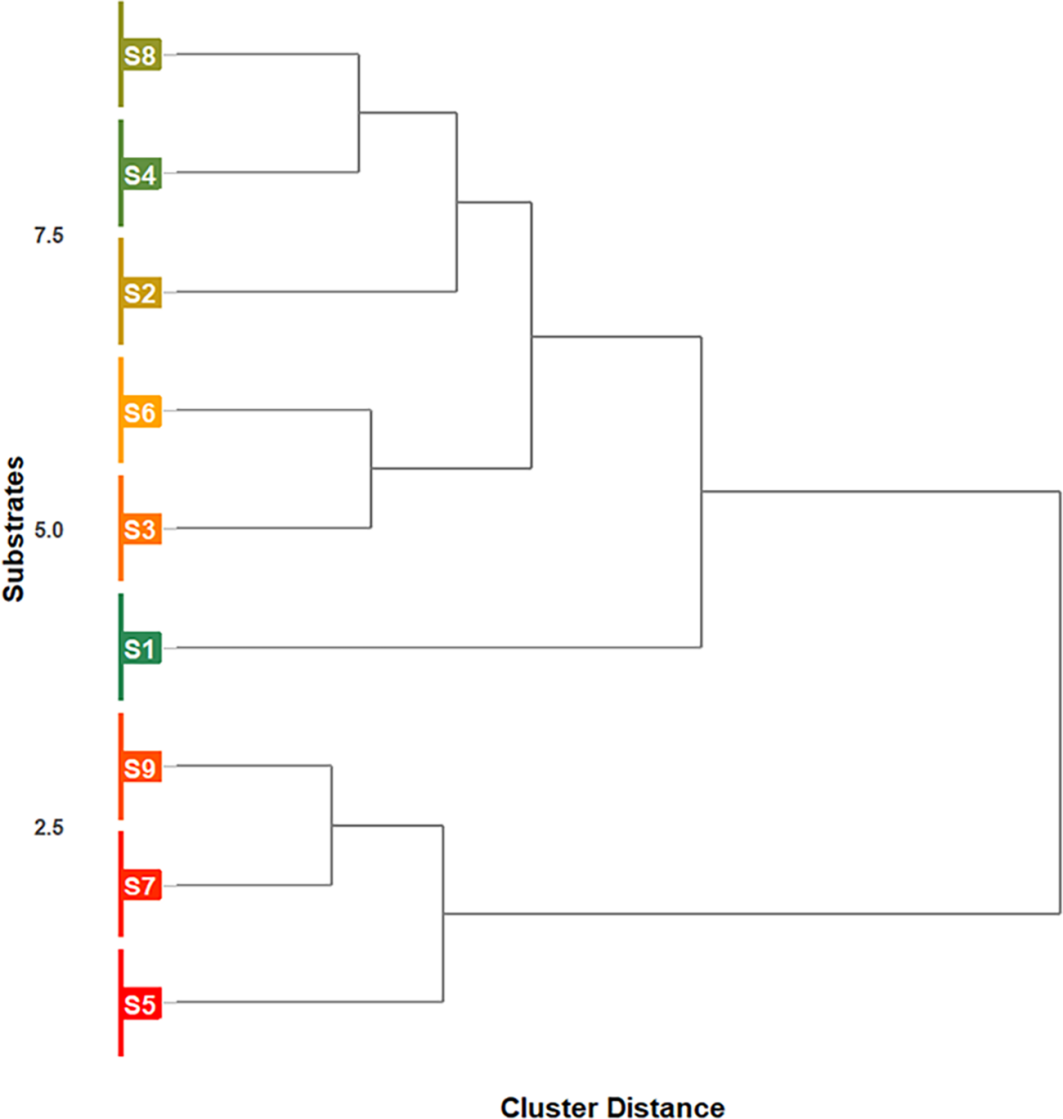
Hierarchical clustering of bell pepper (*C. annuum* L.) seedling performance across nine substrates (S1–S9). The colors indicate the variation in performance of the substrates: green with the development of better-quality seedlings and red with seedlings of inferior phytotechnical standard. S1—Millicompost; S2—Commercial substrate; S3–33% MIL + 33% GLI + 33% PP; S4—33% MIL + 33% GLI + 33% CCF; S5—33% MIL + 33% PP + 33% CCF; S6—25% MIL + 25% GLI + 25% PP + 25% CCF; S7—50% MIL + 50% PP; S8—50% MIL + 50% GLI; S9—50% MIL + 50% CCF. MIL: Millicompost; GLI: *G. sepium*; PP: *P. purpureum*; CCF: commercial coconut fiber powder.

## 4 Discussion

### 4.1 Physical Properties of Organic Substrates

Gonçalves and Poggiani^30^ consider the range of 35–45% to be ideal for macroporosity. Only S1 (43.45%) met this parameter, while the commercial substrate (S2) had macroporosity considered average (27.48%). All the formulated substrates (S3 to S9) had macroporosity below the recommended range, varying from 7.50% to 19.34%. As for microporosity, the authors suggest an ideal range of 45–55%. However, none of the substrates evaluated reached this range. S1 (39.64%) was within the range considered average (25–50%), while the other substrates had high microporosity (>55%), especially S4, S5, S6, and S7, which exceeded 75%. Microporosity is crucial for water retention in the substrate,^31^ and the results obtained corroborate studies which indicate that organic substrates tend to have a predominance of micropores.^32^

Total porosity is a critical factor for plant growth, as it influences oxygen availability and carbon dioxide removal.^33^ The recommended range is 50–80%,^8^ and only S9 (71.27%) fell within this range. The other substrates had total porosity above 80%, with S7 (90.92%) standing out.

With regard to water retention capacity (WRC), Gonçalves and Poggiani^30^ consider values between 20 and 30 mL 50 cm^–3^ to be ideal. In this study, only S9 (30.84 mL 50 cm^–3^) fell within this range. All the other substrates had high WRC (>30 mL 50 cm^–3^), especially S6 (41.18 mL 50 cm^–3^) and S7 (42.67 mL 50 cm^–3^). These results are related to the high microporosity observed in these substrates (Table 2). The higher WRC is an advantage of organic composts, as it reduces the frequency of irrigation.^32^

The volumetric density (VD) of organic substrates should be between 0.30 and 0.75 g cm^–3^,^8^ while Fermino^34^ recommends values of 0.10–0.30 g cm^–3^ for substrates used in trays. In this study, all the substrates, except S1 (0.40 g cm^–3^), showed VD within the recommended range for trays, varying from 0.17 to 0.27 g cm^–3^. S1, which showed a VD 33% above the reference, may have been influenced by the possible addition of soil during its production in the open air, as observed in previous studies.^35^

The VD is an essential parameter for interpreting other substrate properties, such as porosity, aeration and water availability.^34^ In addition, its knowledge is fundamental for irrigation management and nutrient analysis, and is indispensable for interpreting reports and practical recommendations.^36^

### 4.2 Physico-Chemical Properties of Organic Substrates

There are few studies in the literature on the physicochemical characterization of substrates processed by diplopods. Apurva et al.^37^ worked with a substrate processed by *Harpaphe adenina* (Wood) and obtained a pH of 7.20 and an EC of 0.24 dS m^–1^. These differences between the values for pH and electrical conductivities are related to the plant material used in the millicomposting process, since they have varying nutrient contents.^14–16,19^ Kratz et al.^38^ report that for organic substrates without the addition of soil in the composition, the recommendation is in the pH range between 4.4 and 6.2. With the exception of millicompost (S1) and the commercial substrate, the other substrates had pH values above those recommended by the authors, ranging from 6.50 to 9.06 (Table 3).

The raw material that makes up the substrate has a direct influence on pH values. The commercial substrate (S2), which contains pine bark, had a pH of 4.64, corroborating the results obtained by Ludwig et al.^39^ whose values varied between 4.5 and 4.6 in substrates made from pine bark with different granulometries. This more acidic value is characteristic of pine bark, which is naturally acidic.

As for the substrates formulated with millicompost and other raw materials, such as gliricidia, elephant grass and coconut fiber, there was an increase in pH values. For example, substrates S3 (7.90), S4 (7.42), S5 (7.93), S6 (8.19), S7 (8.78) and S8 (9.06) had a more alkaline pH compared to the commercial substrate (S2) (Table 3). This increase can be attributed to the predominance of gliricidia and elephant grass, which naturally have a higher pH. Gliricidia, for example, has a pH value of 9, while elephant grass has a pH value of 11 (Table 3). These high values contributed to the increase in the pH of the formulated substrates and confirm what was observed by Silva et al.^40^ when they evaluated organic composts produced with different proportions of gliricidia and elephant grass.

The predominance of nitrogen in protein form in gliricidia and elephant grass may also have contributed to the alkalization of the substrates. As these plant residues were only air-dried and not composted, there was no acidification induced by the transformation of N-ammonium (NH_4_^+^) into N-nitrate (NO_3_^–^).^41^ In addition, the mineralization of the organic nitrogen present in these raw materials may have favored the production of ammonium (NH_4_^+^), which, together with the low nitrification rate, resulted in a higher pH in substrates S3 to S8 (Table 3).

Electrical conductivity (EC) is an indicator of the concentration of salts in the substrates and is a key parameter for assessing the salinity of the growing medium. High EC values can indicate toxicity to plants, compromising the growth and development of seedlings^42^ Minami and Salvador^43^ point out that EC values above 3.4 dS m^–1^ are considered very high for substrates, values from 2.25 to 3.39 dS m^–1^ are high, values from 1.8 to 2.24 dS m^–1^ are slightly high, values from 0.5 to 1.79 dS m^–1^ are moderate, values between 0.15 and 0.49 dS m^–1^ are low and values below 0.14 dS m^–1^ are considered very low. Thus, most of the substrates had EC in the moderate range (0.5 to 1.79 dS m^–1^), with the exception of the commercial substrate (S2, EC = 0.39 dS m^–1^), which fell into the low range. The EC values ranged from 0.39 dS m^–1^ (S2) to 1.10 dS m^–1^ (S3) (Table 3).

### 4.3 Chemical Properties of Organic Substrates

Nitrogen is the nutrient that influences most of the physiological processes that occur in plants, such as protein synthesis and photosynthesis, and is the most limiting nutrient in the production of biomass,^44^ making it essential in the seedling production phase. In terms of nitrogen content, all the substrates evaluated had levels above the minimum recommended by the aforementioned normative instruction.

Phosphorus has important structural functions for plant development, participating in photosynthesis, respiration, cell division and growth and especially in the supply of energy (ATP), providing greater growth and initial development of plants, especially the root system.^45^ The phosphorus levels considered adequate vary between 0.4 and 0.8 g kg^–1^,^30^ although the available levels were well above this range for all the substrates.

According to Gonçalves and Poggiani,^30^ potassium levels between 1.17 and 3.91 g kg^–1^ are considered adequate. In this case, only substrates S1 (millicompost) and S2 (commercial substrate) were within the range considered adequate and the other substrates had available contents well above the range proposed by the authors, especially S3 and S6. Unlike nitrogen and phosphorus, potassium has no structural function, but it is associated with greater plant resistance when subjected to adverse conditions, such as low water availability and extreme temperatures, due to its function in the opening and closing of stomata,^45^ which did not occur during the development of the bell pepper seedlings.

The levels of calcium considered adequate range from 2 to 4 g kg^–1^ ^30^ and in this study all the substrates had available levels above what is considered adequate. Calcium is a fundamental element in membrane permeability and maintaining cell integrity, and is required for cell division and expansion. It is a component of the cell wall and middle lamella, and also serves as an activator of some enzymes involved in carbohydrate metabolism, such as α-amylase.^46^

As for the levels of available magnesium, all the substrates had Mg contents below the range considered adequate, which according to Gonçalves and Poggiani^30^ ranges from 6 to 12 g kg^–1^. Among the functions of magnesium is its role in the composition of the chlorophyll molecule, participating in various processes such as photosynthesis, respiration, synthesis of carbohydrates and proteins.^47^

The carbon/nitrogen (C/N) ratio helps us to characterize the substrates, indicating how the organic composts are at the end of the composting process,^48^ and it is also indispensable when there are no other types of more robust analyses to ascertain the stability of the compost. Normative Instruction No. 61 of the Ministry of Agriculture, Livestock and Supply^49^ states that the C/N ratio must not exceed 20 and the total nitrogen content must be at least 5.0 g kg^–1^ for organic composts. Therefore, only substrates S3, S7, and S8 had C/N ratios within the recommendations, with ratios below 20.

### 4.4 Morphological Parameters of Bell Pepper Seedlings

Seedling formation is one of the most important stages in vegetable production, as it directly affects the development and production of the crop, influencing its performance in the field.^50^ There are many commercial peat-based substrates on the market and they cost at least BRL 40 per 8 kg (45 L) bag, but the purchase prices end up compromising the producer’s budget. However, the use of organic compost as an alternative to reducing production costs^51^ and promoting sustainability in horticulture,^52^ eliminating the use of peat, besides being an expensive product in countries without local resources,^53^ its extraction not only generates disturbance in the habitats where it occurs and aggravates climate change by releasing carbon reserves.^54^

Raw materials of organic origin are conducive to the development of more vigorous seedlings, because they are able to provide the nutrients needed for growth for various crops.^55,56^ In addition, the use of organic composts acts as a slow-release fertilizer, conserving nutrients such as nitrogen, which are expensive and require a lot of energy to produce.^57,58^

The results of this work showed that the bell pepper seedlings grown in substrates with millicompost showed excellent development, greater dry mass of the shoot and roots, greater height, better vigor and clod stability, reflecting the excellent combination of the physical, physicochemical and chemical properties of this substrate, corroborating results previously recorded with other millicompost formulations based on agricultural and urban waste, in the production of lettuce,^19,16,13^ broccoli (Antunes et al.) and peppers^10^ with superior quality compared to the commercial substrate, indicating that its use is beneficial for the production of seedlings of different horticultural species.

The dry mass of the shoot and roots is a fundamental parameter for assessing the quality and development of vegetable seedlings in substrates. This indicator reflects the vigor of the seedlings, their capacity for growth, establishment and survival after transplanting.^59,60^ Height is also another essential phytotechnical parameter for determining seedling quality. Seedlings with a height of between 15 and 20 cm are considered to be of high quality, reflecting the good initial development made possible by the substrates and, consequently, a greater likelihood of success after transplanting.^61,62,11,13^

Certain combinations of organic materials together with millicompost did not provide better development of the bell pepper seedlings when compared to pure millicompost (S1), and in some cases even hindered their development, since the average values of the dry mass of the shoot and roots, plant height and seedling vigor were lower, as in substrate S5 (33% MIL + 33% PP + 33% CCF). However, combinations of 33% millicompost with 33% gliricidia and 33% coconut fiber (substrate S4) or 50% millicompost + 50% gliricidia (substrate S8) proved to be efficient with average values very close or similar to those of millicompost for dry matter, plant height and seedling vigor.

The cluster analysis and PCA demonstrate that substrate performance is governed by an interdependent balance of physical structure (microporosity and volumetric density) and nutrient availability (K content). This aligns with the growing recognition that sustainable substrate formulation requires multidimensional optimization rather than single-factor adjustments.

Notably, our PCA achieved exceptional explanatory power, with PC1 (78.3%) and PC2 (10.1%) collectively capturing 88.4% of total variance—substantially exceeding the 60–70% threshold required for robust dimensionality reduction.^63,64^ This high cumulative variance underscores the analytical precision of our approach in identifying key performance drivers.

The hierarchical clustering further validated these findings through its strong cophenetic correlation (*r* = 0.89). Such multivariate approaches provide unprecedented resolution for substrate selection, addressing two critical gaps in sustainable horticulture: (1) the traditional overreliance on single-parameter optimization, and (2) the lack of quantitative frameworks aligning substrate properties with SDG-compliant production (specifically SDG 2.4 [sustainable food production and resilient agricultural practices] and 12.2 [sustainable management and efficient use of natural resources]).

Our methodology offers a novel decision-making tool where substrates can be systematically evaluated not just by individual growth parameters, but through their integrated performance across physical, chemical, and biological axes. This represents a paradigm shift from empirical formulations to data-driven substrate design, particularly valuable for developing circular growing media from agroindustrial byproducts.

The different substrate formulations exhibited distinct chemical properties due to the intrinsic characteristics of their raw materials. As organic substrates, their nutrient release dynamics differ from mineral-based substrates, which typically provide readily available nutrients. The phytotechnical performance of seedlings depends on the interplay of physical, physicochemical, and chemical properties, as evaluated in this study. For bell pepper seedlings in their initial growth phase, most substrate formulations adequately provided nutrients, except for S5 (33% MIL + 33% PP + 33% CCF), which showed poor performance across all measured parameters.

## 5 Conclusions

The formulations of different organic substrates allowed us to verify that the production of bell pepper seedlings is effective when millicompost, gliricidia and coconut fiber are used in the proportion of 33% of each material (substrate S4) or in the formulation of millicompost and gliricidia with 50% of each component (substrate S8).

The other formulations produce seedlings with regular vigor, except for substrate S5 (33% MIL + 33% PP + 33% CCF). However, it is suggested that these formulations be tested with other vegetable species in order to determine whether the responses to plant development are better or not.

Combining millicompost with other organic waste that is readily available to the producer makes it possible to maximize its use on the farm, allowing quality seedlings to be generated with a smaller quantity of compost, as well as being a sustainable alternative that replaces the use of peat and other raw materials that impact the environment.
